# CracidMex1: a comprehensive database of global occurrences of cracids (Aves, Galliformes) with distribution in Mexico

**DOI:** 10.3897/zookeys.420.7050

**Published:** 2014-06-25

**Authors:** Gonzalo Pinilla-Buitrago, Miguel Angel Martínez-Morales, Fernando González-García, Paula L. Enríquez, José Luis Rangel-Salazar, Carlos Alberto Guichard Romero, Adolfo G. Navarro-Sigüenza, Tiberio César Monterrubio-Rico, Griselda Escalona-Segura

**Affiliations:** 1 El Colegio de la Frontera Sur, unidad Campeche. Avenida Rancho Polígono 2-A, Ciudad Industrial, Lerma, Campeche, Campeche, 24500, Mexico; 2 Red de Biología y Conservación de Vertebrados, Instituto de Ecología, AC. Carretera antigua a Coatepec No. 351, El Haya, Xalapa, Veracruz, 91070, Mexico; 3 El Colegio de la Frontera Sur, unidad San Cristóbal. Carretera Panamericana y Periférico Sur s/n, Barrio María Auxiliadora, San Cristóbal de Las Casas, Chiapas, 29290, Mexico; 4 Zoológico Miguel Álvarez del Toro. Calzada Cerro Hueco s/n, Colonia Zapotal, Apartado Postal 6, Tuxtla Gutiérrez, Chiapas, 29094, Mexico; 5 Museo de Zoología, Facultad de Ciencias, Universidad Nacional Autónoma de México. Apartado Postal 70-399, México DF, 04510, Mexico; 6 Facultad de Biología, Universidad Michoacana de San Nicolas de Hidalgo. Edificio R, Ciudad Universitaria, Morelia, Michoacán, 58000, Mexico; 7 Present address: Instituto de Ciencias Naturales, Universidad Nacional de Colombia. Ciudad Universitaria, Av. Carrera 30 No. 45, Bogotá DC, 111321, Colombia

**Keywords:** *Ortalis*, *Penelope*, *Penelopina*, *Oreophasis*, *Crax*, Cracidae, Aves, chachalacas, guans, curassows, Mexico, Neotropic, geographic record, Darwin Core

## Abstract

Cracids are among the most vulnerable groups of Neotropical birds. Almost half of the species of this family are included in a conservation risk category. Twelve taxa occur in Mexico, six of which are considered at risk at national level and two are globally endangered. Therefore, it is imperative that high quality, comprehensive, and high-resolution spatial data on the occurrence of these taxa are made available as a valuable tool in the process of defining appropriate management strategies for conservation at a local and global level. We constructed the CracidMex1 database by collating global records of all cracid taxa that occur in Mexico from available electronic databases, museum specimens, publications, “grey literature”, and unpublished records. We generated a database with 23,896 clean, validated, and standardized geographic records. Database quality control was an iterative process that commenced with the consolidation and elimination of duplicate records, followed by the geo-referencing of records when necessary, and their taxonomic and geographic validation using GIS tools and expert knowledge. We followed the geo-referencing protocol proposed by the Mexican National Commission for the Use and Conservation of Biodiversity. We could not estimate the geographic coordinates of 981 records due to inconsistencies or lack of sufficient information in the description of the locality.

Given that current records for most of the taxa have some degree of distributional bias, with redundancies at different spatial scales, the CracidMex1 database has allowed us to detect areas where more sampling effort is required to have a better representation of the global spatial occurrence of these cracids. We also found that particular attention needs to be given to taxa identification in those areas where congeners or conspecifics co-occur in order to avoid taxonomic uncertainty. The construction of the CracidMex1 database represents the first comprehensive research effort to compile current, available global geographic records for a group of cracids. The database can now be improved by continuous revision and addition of new records. The CracidMex1 database will provide high quality input data that could be used to generate species distribution models, to assess temporal changes in species distributions, to identify priority areas for research and conservation, and in the definition of management strategies for this bird group. This compilation exercise could be replicated for other cracid groups or regions to attain a better knowledge of the global occurrences of the species in this vulnerable bird family.

## Introduction

Cracids are a primitive family of Neotropical Galliformes. They are mainly frugivorous birds that inhabit primary forests, and may play an important role in regenerating and structuring forests through the dispersion and predation of seeds (Peres and Roosmalen 1996; Sedaghatkish 1996; Muñoz and Kattan 2007). Based on this and on their sensitivity to disturbance, the presence of viable populations of cracids in an area is considered indicative of forest quality.

Cracids are one of the most vulnerable groups of Neotropical birds because almost half of the 54 recognized species (AOU 2014) are at risk, and some of them are almost extinct (Brooks and Strahl 2000). This vulnerability is a consequence of their strong dependence on primary forests, and their susceptibility to habitat destruction and degradation, in addition to the intensity of hunting faced by cracids (Silva and Strahl 1991, 1997; Brooks and Strahl 2000; del Hoyo and Motis 2004). These factors together with life history traits of delayed age of first reproduction, low chick survival, and low reproduction rates, act in synergy to exacerbate the vulnerability of cracids to human pressures. In Mexico there are 12 cracid taxa of which six are included in the national list of threatened species (SEMARNAT 2010) and two (*Oreophasis derbianus* and *Crax rubra griscomi*) are globally endangered (Brooks and Strahl 2000; Martínez-Morales et al. 2009; IUCN 2013).

The lack of up to date, high quality data on the presence and abundance of cracids in many regions of their distribution prevents the definition and implementation of appropriate management strategies for their conservation (Brooks and Strahl 2000; González-García et al. 2001). Although their distribution has already been depicted in maps (Delacour and Amadon 2004; Ridgley et al. 2012), and even analysed in the context of global climate change (Peterson et al. 2001), we still do not know the present species distribution with a high level of certainty as a result of continual changes in forest cover. Not to mention that for several species or regions there are still significant gaps in knowledge of species distribution. In this regard, the former Cracid Specialist Group recommended an urgent revision of cracid distribution (Brooks and Strahl 2000; Brooks 2006).

To tackle this imperative need for information, we constructed the CracidMex1 database that embodies an exhaustive, high quality, and updated compilation of the global geographic records of the eight cracid species with distribution in Mexico. The collation of records from numerous sources required a thorough process of quality control in terms of consolidation and elimination of record redundancies, completion of missing data, verification of record localities and their spatial precision, and validation of taxa identity. This involved an iterative process of automatized tasks and the use of expert knowledge in terms of species and regions.

The CracidMex1 database will provide high quality, input data that could be used to identify areas where more research is needed, generate species distribution models, assess temporal changes in species distribution, identify priority areas for cracid conservation, and even in the definition of management strategies for this avian group. This compilation exercise could be replicated for other groups of cracids or regions to achieve a more complete knowledge of the global occurrences of the species of this vulnerable bird family.

This open access database will be continuously reviewed and supplemented with additional records, and all contributions to the database are very welcome.

## Data published through

http://ipt.pensoft.net/ipt/resource.do?r=cracidmex1

## Taxonomic ranks

**Kingdom:** Animalia

**Phylum:** Chordata

**Class:** Aves

**Order:** Galliformes

**Family:** Cracidae

**Genera:** *Ortalis*, *Penelope*, *Penelopina*, *Oreophasis*, *Crax*

**Species:** *Ortalis vetula* (Wagler, 1830), *Ortalis wagleri* Gray, 1867, *Ortalis poliocephala* (Wagler, 1830), *Ortalis leucogastra* (Gould, 1843), *Penelope purpurascens* Wagler, 1830, *Penelopina nigra* (Fraser, 1852), *Oreophasis derbianus* Gray, 1844, *Crax rubra* Linnaeus, 1758 (Table 1).

**Table 1.** Conservation and endemic features of the cracid taxa included in the CracidMex1 database.

Species/Subspecies	Common name	CITES^1^	IUCN^2^	NOM-059^3^	Endemicity
*Ortalis vetula*	Plain Chachalaca	III^4^	Least Concern		Not endemic
*Ortalis vetula vetula*					• E Mexico to Costa Rica
*Ortalis vetula mccalli*					• SE USA, E Mexico
*Ortalis vetula pallidiaventris*					• Yucatan Peninsula (Mexico)
*Ortalis vetula deschauenseei*					• Utila Island (Honduras)
*Ortalis vetula intermedia*					• S Mexico, Guatemala, Belize
*Ortalis wagleri*	Rufous-bellied Chachalaca		Least Concern		North western Mexico
*Ortalis poliocephala*	West Mexican Chachalaca		Least Concern		Central western Mexico
*Ortalis leucogastra*	White-bellied Chachalaca		Least Concern	Special protection	Northern Central America (Pacific slope)
*Penelope purpurascens*	Crested Guan	III^5^	Least Concern	Threatened	Not endemic
*Penelope purpurascens purpurascens*				• Threatened	• Not endemic
*Penelope purpurascens aequatorialis*					• Not endemic
*Penelope purpurascens brunnescens*					• N Colombia, N Venezuela
*Penelopina nigra*	Highland Guan	III^6^	Vulnerable	Endangered	Northern Central America
*Oreophasis derbianus*	Horned Guan	I	Endangered	Endangered	S Mexico, Guatemala
*Crax rubra*	Great Curassow	III^7^	Vulnerable	Threatened	Not endemic
*Crax rubra rubra*				• Threatened	• Not endemic
*Crax rubra griscomi*				• Endangered	• Cozumel Island (Mexico)

^1^ Convention on International Trade in Endangered Species of Wild Fauna and Flora <http://www.cites.org/eng/app/appendices.php>.

^2^ The IUCN Red List of Threatened Species <http://www.iucnredlist.org>.

^3^ Mexican environmental legislation (SEMARNAT 2010).

^4^ Guatemala and Honduras.

^5^ Honduras.

^6^ Guatemala.

^7^Guatemala, Honduras, Costa Rica, and Colombia.

**Common names:** Chachalacas, Guans, and Curassows

## Taxonomic coverage

**Figure 1.**
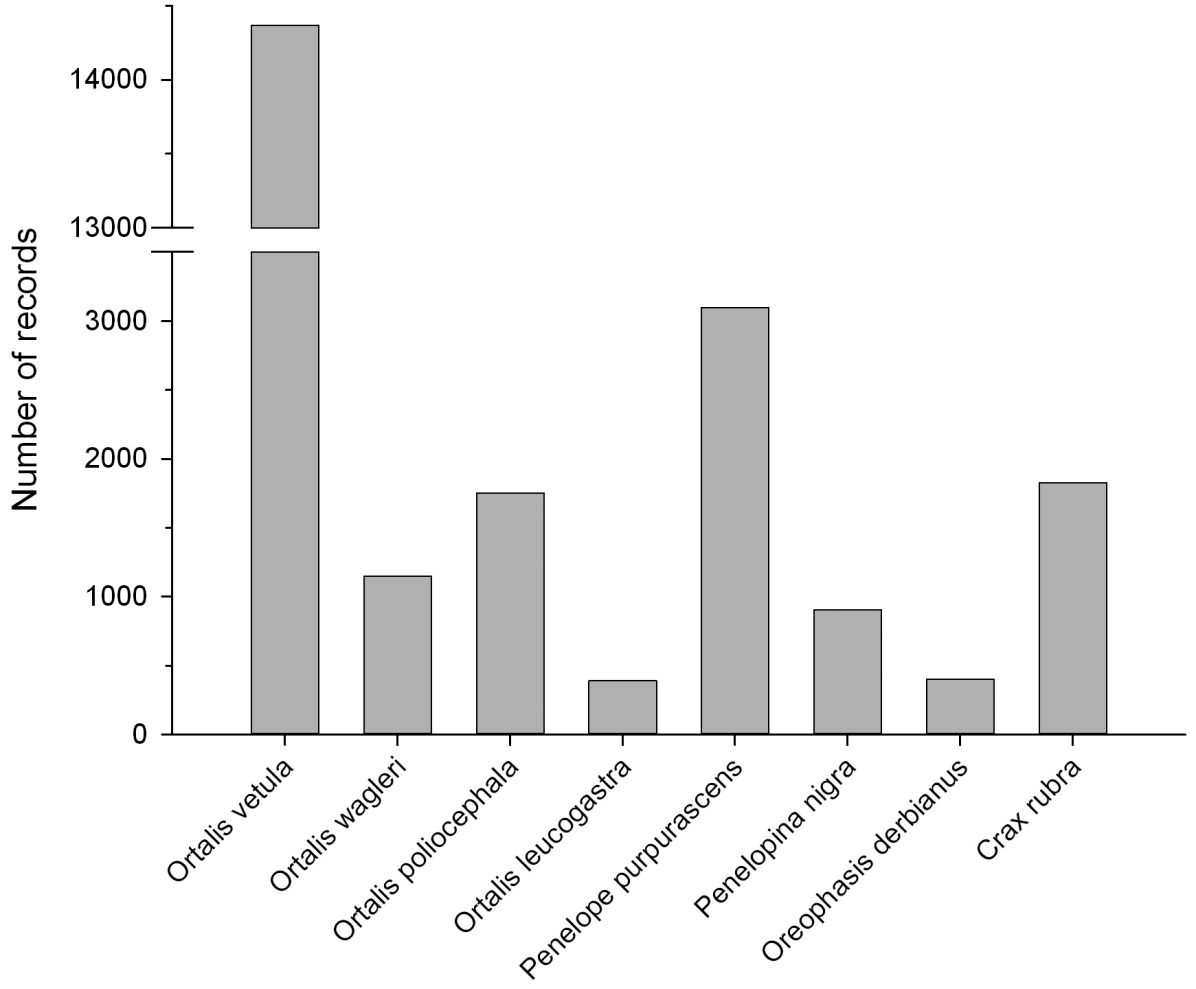
Distribution of the 23,896 records by species in the CracidMex1 database.

The CracidMex1 database comprises 23,896 global records of 12 taxa of cracid species and subspecies with distribution in Mexico. This includes eight cracid species distributed in Mexico, out of the 54 recognized species in the Neotropical region (AOU 2014). The database also includes records of *Ortalis vetula deschauenseei* from the Utila Island, Honduras, and of two other subspecies of *Penelope purpurascens* (*aequatorialis* and *brunnescens*) which are not distributed in Mexico (Table 2). The genus *Ortalis* accounted for most of the records, followed by *Penelope*, *Crax*, *Penelopina*, and *Oreophasis*. This bias in records at a genus level is also mirrored at species level (Figure 1). However, at subspecies level this bias is not evident because only 19.9% of the records assignable to subspecies level are given to this taxonomic level (4.6% in *Ortalis vetula*, 43.5% in *Penelope purpurascens*, and 100% in *Crax rubra*).

**Table 2.** Number of records in the CracidMex1 database by genus, species, and subspecies.

Genus/Species/Subspecies	Records	Proportion (%)
*Ortalis*	17,663	73.9
*Ortalis vetula*	14,366	60.1
*Ortalis vetula vetula*	193	0.8
*Ortalis vetula mccalli*	291	1.2
*Ortalis vetula pallidiventris*	119	0.5
*Ortalis vetula deschauenseei*	4	0.0
*Ortalis vetula intermedia*	58	0.2
*Ortalis wagleri*	1,151	4.8
*Ortalis poliocephala*	1,754	7.3
*Ortalis leucogastra*	392	1.6
*Penelope*	3,100	13.0
*Penelope purpurascens*	3,100	13.0
*Penelope purpurascens purpurascens*	1,152	4.8
*Penelope purpurascens aequatorialis*	164	0.7
*Penelope purpurascens brunnescens*	29	0.1
*Penelopina nigra*	907	3.8
*Oreophasis derbianus*	401	1.7
*Crax*	1,825	7.6
*Crax rubra*	1,825	7.6
*Crax rubra rubra*	1,797	7.5
*Crax rubra griscomi*	28	0.1

### Spatial coverage

**General spatial coverage**

**Figure 2.**
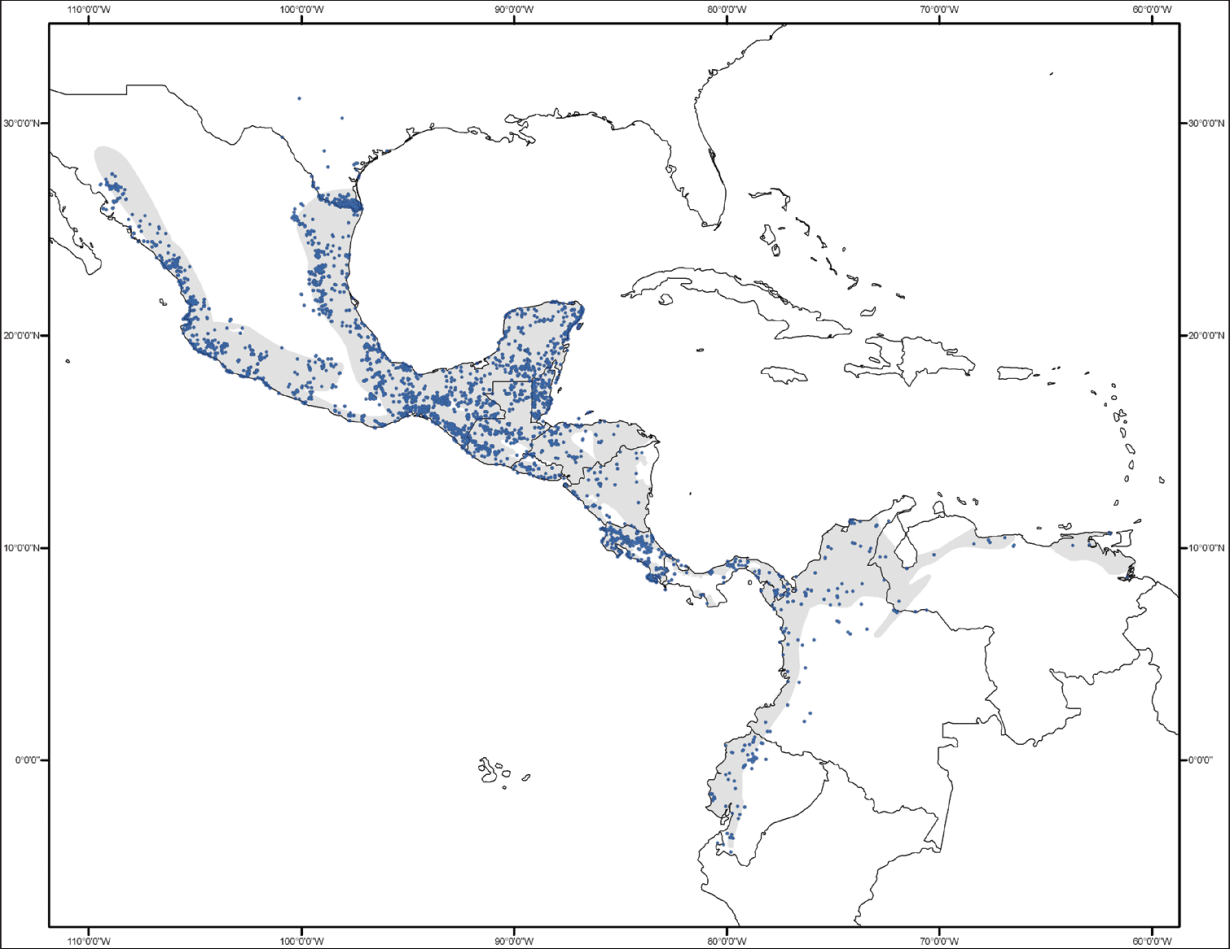
Geographic distribution of the 22,731 valid distributional records of cracids in the CracidMex1 database. Grey shadeing represents the area where the species occurrence is expected based on Ridgley et al. (2012).

**Figure 3.**
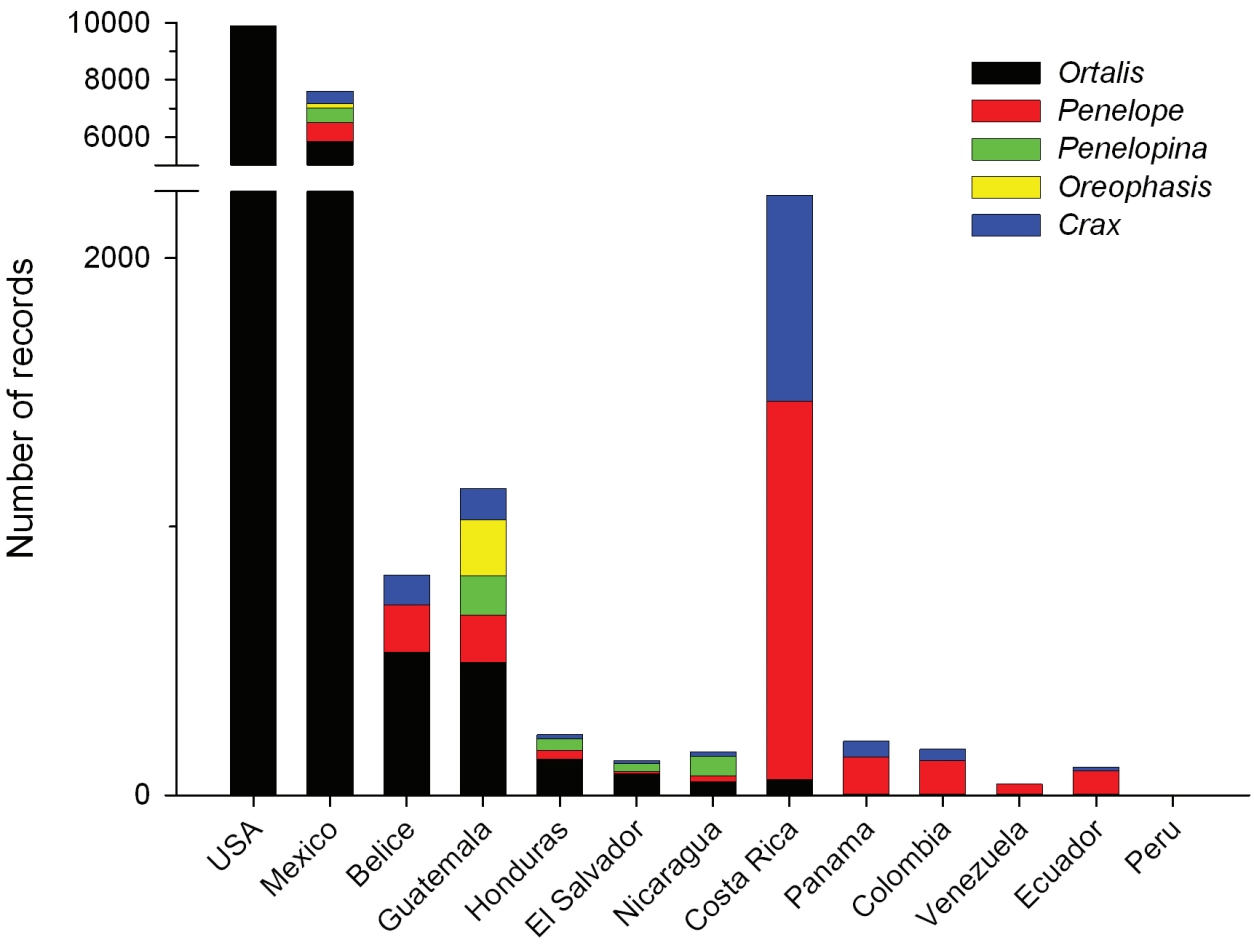
Distribution of cracid genera by country for the 22,731 valid distributional records in the CracidMex1 database.

Valid distributional records (22,731), based on the native distribution of taxa, cover distributions from southern Texas, USA, in the north, to Loja, Ecuador, in the south, including Mexico, Belize, Guatemala, Honduras, El Salvador, Nicaragua, Costa Rica, Panama, Colombia, Venezuela, and Peru (Table 3, Figures 2 and 3). These records are labelled as *presente* (present) in the “occurrenceStatus” field of the database. Other records corresponded to zoo specimens (49), records with spatial inconsistencies or ambiguities (143), and records for which coordinates could not be calculated due to insufficient information in the description of the locality (981). These records are labelled as *ausente* (absent) or *dudoso* (doubtful) in the “occurrenceStatus” field. In this case a label of “absent” (186 records) means that the record is out of the distributional range of the species (e.g., zoo records), and “doubtful” (979) means that the species could be present in the area, but the ambiguity in the description of the locality prevents an unequivocal assertion of the spatial validity of the record (e.g., Locality: Mexico).

**Table 3.** Number of valid distributional records of cracid species by country in the CracidMex1 database.

Country	*Ortalis vetula*	*Ortalis wagleri*	*Ortalis poliocephala*	*Ortalis leucogastra*	*Penelope purpurascens*	*Penelopina nigra*	*Oreophasis derbianus*	*Crax rubra*	Total
USA	9,904	0	0	0	0	0	0	0	9,904
Mexico	2,938	1,113	1,675	124	642	533	145	430	7,600
Belize	533	0	0	0	175	0	0	112	820
Guatemala	408	0	0	87	176	145	210	115	1,141
Honduras	134	0	0	0	33	42	0	16	225
El Salvador	1	0	0	78	10	29	0	10	128
Nicaragua	17	0	0	33	21	73	0	17	161
Costa Rica	57	0	0	0	1,410	0	0	769	2,236
Panama	0	0	0	0	141	0	0	59	200
Colombia	0	0	0	0	128	0	0	43	171
Venezuela	0	0	0	0	41	0	0	0	41
Ecuador	0	0	0	0	90	0	0	13	103
Peru	0	0	0	0	1	0	0	0	1
Total	13,992	1,113	1,675	322	2,868	822	355	1,584	22,731

**Coordinates**

-4.3327 to 31.1707 Latitude; -109.4433 to -61.1382 Longitude. This range includes the location of only the 22,731 valid distributional records (Figure 2).

## Temporal coverage

**Figure 4.**
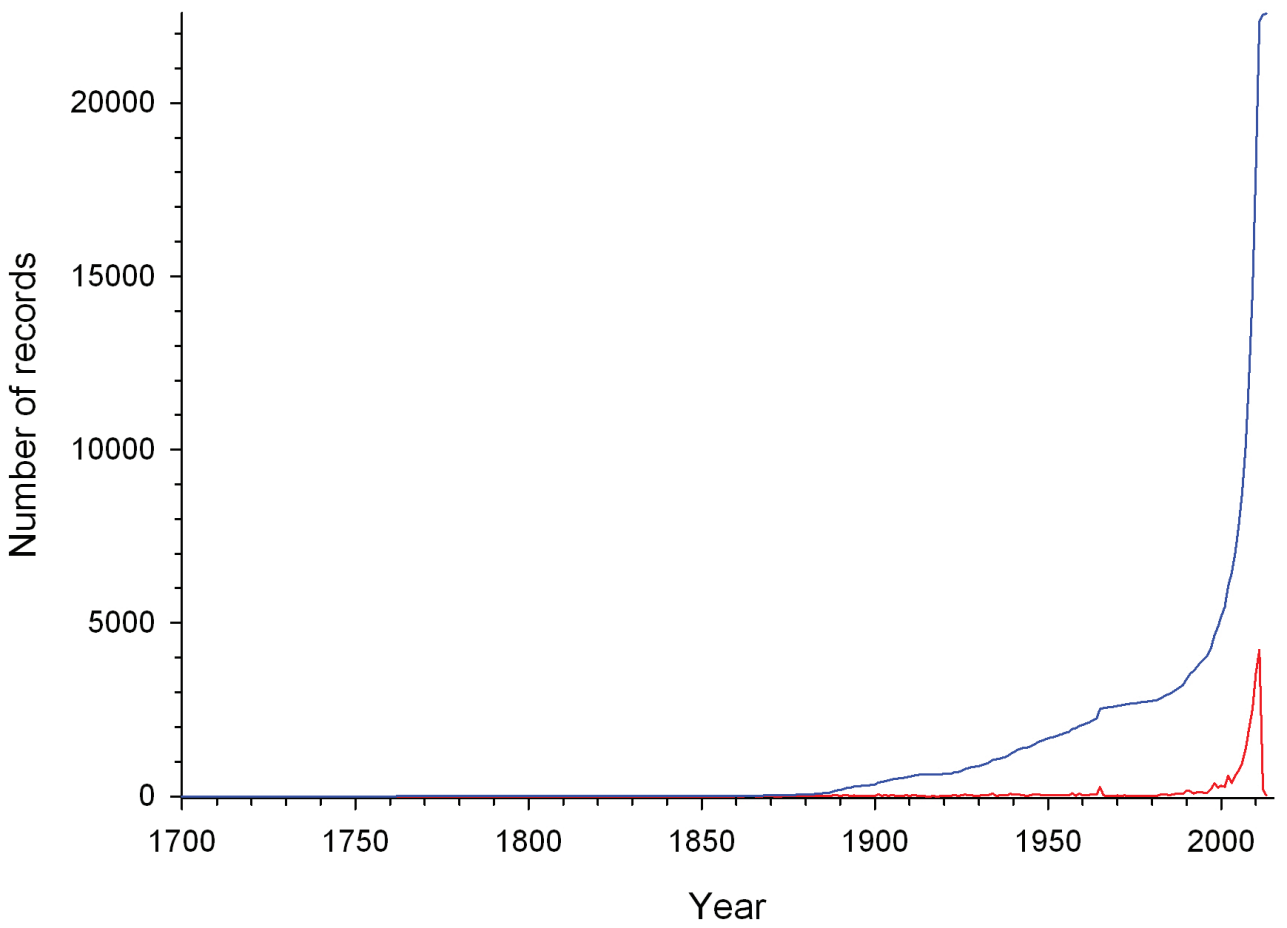
Number of cracid records gathered per year (red line) and the cumulative number of cracid records gathered from 1700 to 2013 (blue line).

The date of occurrence records (year-month-day) encompasses from 1700-01-01 to 2013-10-25. However, of the 22,731 valid distributional records, 854 lack information on recording date. Although temporal coverage spans more than 300 years, most of the records were generated in the last decades (Figure 4). A boom in reporting or generating species records started at the end of the last century, most probably due to the emergence of the Internet and technological advancement in field survey equipment. Additionally, this observed pattern might be due to an increased interest in studying this bird group. Information gathered through years of research and observation of the species’ natural history led to the publication in 1973 of the first edition of the inspiring book “Curassows and related birds” by Delacour and Amadon. Added to which the First International Symposium on the Family Cracidae was organized in 1981, which may also have triggered an exponential increase in the interest for studying this avian group, and thus, an increase in reporting species occurrences.

## Project description

**Title:** Present and future distribution models of cracids occurring in Mexico.

**Personnel:** Miguel Angel Martínez-Morales (Project Coordinator, Resource Contact, Resource Creator), Gonzalo Enrique Pinilla-Buitrago (Database Manager, Metadata Provider), Fernando González-García, Paula L. Enríquez, José Luis Rangel-Salazar, Carlos Alberto Guichard Romero, Adolfo G. Navarro-Sigüenza, Tiberio César Monterrubio-Rico, Griselda Escalona-Segura (Data Contributors).

**Funding:** National Commission for Knowledge and Use of Biodiversity (CONABIO), Mexico, under the agreement FB1585/JM024/12.

**Figure 5.**
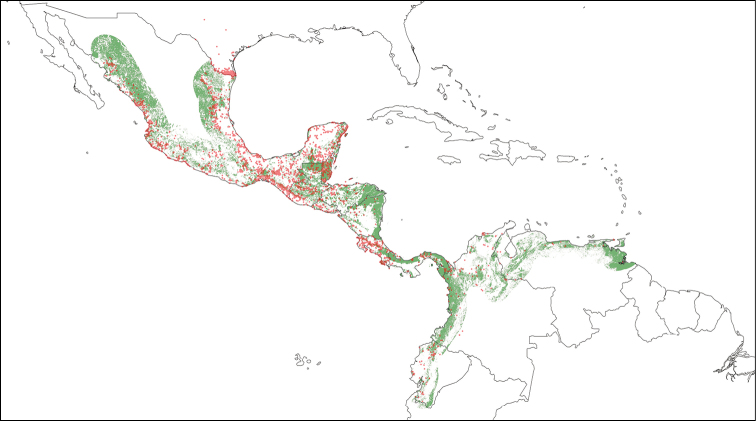
Geographic distribution of the 22,731 valid distributional records of cracids in the CracidMex1 database. Present pattern of forest cover is depicted in green shading. Forest cover was obtained from INEGI (2012) for Mexico, the World Bank and CCAD (2000) for Central America, and the European Commission Joint Research Centre (http://www-gem.jrc.it/glc2000) for South America.

**Study area descriptions/descriptor:** Valid distribution records are located in the northern portion of the Neotropical region, including the transitional zone with the Nearctic region (Figure 5). Native vegetation in this area ranges from tropical dry to humid forests, and from lowlands to montane forests. However, a large proportion of the native vegetation has been converted to pasture and agricultural areas. The expansion of human settlements, infrastructure, and mining have also contributed to forest degradation and deforestation in the region. Tropical forests have the largest net loss of forested area compared to other forest types in the world (FAO and JRC 2012), and the Neotropical region is not the exception. The study area includes the Mesoamerica biodiversity hotspot, the Chocó/Darién/Western Ecuador hotspot, and marginally the Tropical Andes hotspot (Myers et al. 2000), but these hotspots harbour only 20 to 25% of the original extent of primary vegetation.

Given the current pattern of forest cover in the region, and the temporal coverage of records in the CracidMex1 database, many records, particularly older records, are now located outside of currently forested areas (Figure 5). This suggests a substantial reduction in the distribution of cracid species, particularly for those species restricted to primary forests (*Penelope purpurascens*, *Penelopina nigra*, *Oreophasis derbianus*, and *Crax rubra*). Habitat loss and hunting pressure are the main drivers of cracid population declines and distribution contractions, the synergy of which has caused the endangerment of these species (Silva and Strahl 1991, 1997; Brooks and Strahl 2000; del Hoyo and Motis 2004).

**Design description:** The construction of the CracidMex1 database aimed to gather most of the globally available records of cracids which are distributed in Mexico, in order to generate global species distribution models. We initiated the construction of the database by collating records from six electronic databases available through the Internet: GBIF <http://data.gbif.org>, ORNIS <http://www.ornisnet.org>, REMIB <http://www.conabio.gob.mx/remib/doctos/remib_esp.html>, UNIBIO <http://unibio.unam.mx>, SpeciesLink <http://splink.cria.org.br>, and IBC <http://ibc.lynxeds.com>. Additionally, we obtained records from the National System of Information on Biodiversity (SNIB) database at CONABIO and from museum specimen records contained in the Bird Atlas of Mexico database at the Facultad de Ciencias of the National Autonomous University of Mexico. We also obtained records from published papers through searches in BioOne <http://www.bioone.org>, EBSCO <http://search.ebscohost.com>, JSTOR <http://www.jstor.org>, ScienceDirect <http://www.sciencedirect.com>, Springer Link <http://www.springerlink.com>, Web of Science <http://apps.webofknowledge.com>, Wiley Online Library <http://onlinelibrary.wiley.com>, Zoological Record <http://thomsonreuters.com/zoological-record/>, Redalyc <http://www.redalyc.org>, SciELO <http://www.scielo.org>, and Google Scholar <scholar.google.com>. We also reviewed the bulletins of the Cracid Group of the Galliformes Specialists Group <http://www.cracids.org>. Added to which, we gathered records from “grey literature” through searches in technical reports and theses. These searches included the electronic portal of CONABIO and the repositories OpenDOAR <http://opendoar.org> and the Registry of Open Access Repositories <http://roar.eprints.org>. Finally, we gathered records from our own and unpublished databases of colleagues through personal contacts. After the GBIF, these personal unpublished databases were the second most important source of records, followed by records gathered from the SNIB and published papers (Table 4).

**Table 4.** Relative contribution of records of cracid species by the different sources used in the construction of the CracidMex1 database. Numbers represent non-duplicate records. GBIF was the main source of records, but its relative contribution is magnified in this table because in the consolidation process we considered this source as the reference database.

Source	*Ortalis vetula*	*Ortalis wagleri*	*Ortalis poliocephala*	*Ortalis leucogastra*	*Penelope purpurascens*	*Penelopina nigra*	*Oreophasis derbianus*	*Crax rubra*	Total
GBIF	13,479	982	896	279	2,751	734	233	1,524	20,878
ORNIS	180	19	11	64	2	1	0	2	279
REMIB	86	0	0	0	0	0	0	12	98
UNIBIO	0	0	0	0	0	0	0	0	0
SpeciesLink	1	0	0	0	5	0	0	1	7
IBC	0	0	0	0	1	0	0	1	2
SNIB	209	1	435	8	17	26	9	12	717
Bird Atlas Mex	120	95	31	1	57	34	2	51	391
Published papers	235	47	77	37	131	56	40	90	713
“Grey literature”	37	3	6	3	20	4	2	16	91
Unpublished DB	19	4	298	0	116	52	115	116	720
Total	14,366	1,151	1,754	392	3,100	907	401	1,825	23,896

**Figure 6.**
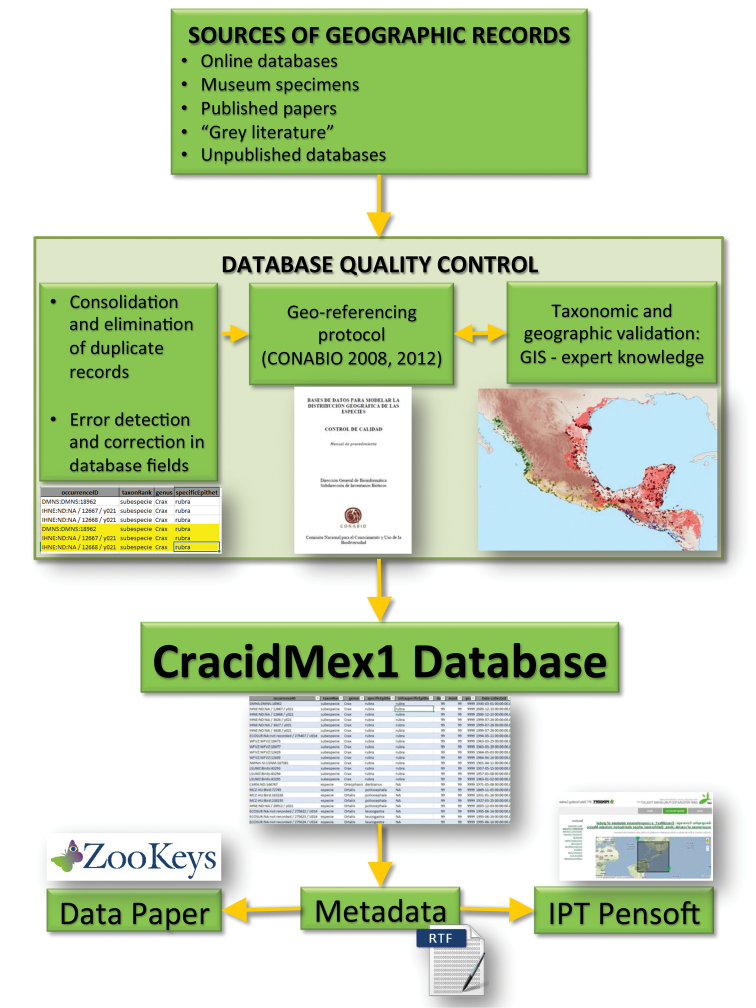
Flowchart depicting the iterative process for the construction of the CracidMex1 database up to publication.

Database quality control, based on the standards described in CONABIO (2012), was an iterative process that commenced with the detection, consolidation and elimination of duplicate records (the same record reported in more than one source). For detection of duplicate records within and among sources we first gave priority to the fields “institutionCode”, “catalogNumber”, “country”, “state”, “locality”, “decimalLatitude”, and “decimalLongitude”. The consolidation process consisted of the creation of a single record with more complete data from duplicate records. In the case of inconsistencies in duplicate records, we referred to the original source of the record. We avoided and corrected errors (omission, typographic, contextual, redundancy, convention, and congruence) through automatized tasks and case by case revision of the database. We then calculated geographic coordinates and their uncertainties for those records lacking these data, based on the standards described in CONABIO (2008). All coordinates refer to the datum WGS84. We used a variety of resources for geo-referencing, namely Google Earth 7 <http://www.google.com/earth/index.html>, Google Maps and the tools of Map Labs <http://maps.google.com>, glosk <http://www.glosk.com/>, CONABIO <http://www.conabio.gob.mx/informacion/metadata/gis/loc2000gw.xml?_httpcache=yes&_xsl=/db/metadata/xsl/fgdc_html.xsl&_indent=no>, GEOSiB <http://www.humboldt.org.co/geoinformacion/geosib>, and Georeferencing Calculator <http://manisnet.org/gci2.html>. We also consulted regional experts for advice during the geo-referencing process. Once we were sufficiently certain of the correct location of the record, we checked that each location was consistent with taxa identification by displaying the records in a GIS. This taxonomic and geographic validation through the use of GIS tools and expert knowledge allowed us to detect inconsistencies. Where possible, we corrected inconsistencies through an iterative process, otherwise we labelled the record as “doubtful” (979 records) or “absent” (186) in the “occurrenceStatus” field as described above (Figure 6).

The CracidMex1 database has 41 fields based on the standard Darwin Core version 1.4 (Table 5).

**Table 5.** Definition of fields included in the CracidMex1 database based on the standard Darwin Core version 1.4.

Field	Definition
institutionCode	The name (or acronym) in use by the institution having custody of the object(s) or information referred to in the record. In the case of personal records, we used the value “NA” *No aplica* (Not applicable).
collectionCode	The name, acronym, code, or initials identifying the collection or data set from which the record was derived. If the record was not held in a collection, we used the value “NA” *No aplica* (Not applicable). If the collection name was not known, we used the value “ND” *No determinado* (Not determined).
datasetName	The name identifying the data set from which the record was derived. If the data set name was not known, we used the value “ND” *No determinado* (Not determined).
basisOfRecord	The specific nature of the data record. • *Ejemplar preservado* (Preserved specimen). Denoting a preserved specimen in a collection. • *Observación* (Human observation). Denoting an observation made by one or more people. • *Observación con aparato* (Machine observation). Denoting an observation made by a machine. • *Ocurrencia* (Occurrence). Denoting a case where no information is available on how the record was obtained.
occurrenceID	A uniform resource name as a unique identifier for the record. In the absence of a persistent global unique identifier, this was constructed in the form: “[institutionCode]: [collectionCode]: [catalogNumber]”. If the record lacked a value in one of these fields (NA or ND) a sequential number was assigned at the end.
catalogNumber	An identifier for the record within the data set or collection. If the record did not have a catalogue number, we used the value “NA” *No aplica* (Not applicable). If we did not know the catalogue number, we used the value “ND” *No determinado* (Not determined).
recordNumber	An identifier given to the occurrence at the time it was recorded. This often serves as a link between field notes and an occurrence record, such as a specimen collector’s number. If the record did not have a record number, we used the value “NA” *No aplica* (Not applicable). If we did not know the record number, we used the value “ND” *No determinado* (Not determined).
recordedBy	A list (concatenated and separated) of names of people, groups, or organizations responsible for recording the original occurrence. The primary collector or observer, especially one who applies a personal identifier (recordNumber), is listed first. If we did not know the name of the collector, we used the value “ND” *No determinado* (Not determined).
individualCount	The number of individuals recorded at the time of the occurrence. We left the value empty if individualCount was unknown.
occurrenceStatus	A statement about the presence or absence of a taxon at a location. • *Presente* (Present). There is at least one well documented record of the taxon’s presence in the area. • *Ausente* (Absent). There is evidence to document the absence of a taxon in the area. • *Dudoso* (Doubtful). The taxon is presumed present in the area, but there is doubt over the evidence, including taxonomic or geographic imprecision in the records.
associatedReferences	A list (concatenated and separated) of identifiers (publication, bibliographic reference, global unique identifier) of literature associated with the occurrence. If no reference was associated, we used the value “NA” *No aplica* (Not applicable).
year	The four-digit year in which the event occurred, according to the Common Era Calendar. If we did not know the year, we used “9999”.
month	The ordinal month in which the event occurred. If we did not know the month, we used “99”.
day	The integer day of the month on which the event occurred. If we did not know the day, we used “99”.
country	The name of the country or major administrative unit in which the location occurs. If we did not know the name, we used the value “ND” *No determinado* (Not determined).
stateProvince	The name of the next smaller administrative region below country (state, province, canton, department, region, etc.) in which the location occurs. If we did not know the name, we used the value “ND” *No determinado* (Not determined).
county	The full, unabbreviated name of the next smaller administrative region below stateProvince (county, shire, department, municipality) in which the location occurs. If this administrative region does not apply, we used the value “NA” *No aplica* (Not applicable). If we did not know the name, we used the value “ND” *No determinado* (Not determined).
locality	The specific description of the place. This term may contain information modified from the original to correct perceived errors or standardize the description. If we did not know the description, we used the value “ND” *No determinado* (Not determined).
decimalLatitude	The geographic latitude (in decimal degrees, using the spatial reference system given in geodeticDatum) of the geographic centre of a location. Positive values are north and negative values are south of the Equator. We left the value empty if decimalLatitude was unknown.
decimalLongitude	The geographic longitude (in decimal degrees, using the spatial reference system given in geodeticDatum) of the geographic centre of a location. Positive values are east and negative values are west of the Greenwich Meridian. We left the value empty if decimalLongitud was unknown.
geodeticDatum	The ellipsoid, geodetic datum, or spatial reference system upon which the geographic coordinates given in decimalLatitude and decimalLongitude are based. We used the value “ND” *No determinado* (Not determined) when no data was available in decimalLatitude and decimalLongitude.
coordinateUncertaintyInMeters	The horizontal distance (in meters) from the given decimalLatitude and decimalLongitude describing the smallest circle containing the entire location. We left the value empty if the uncertainty was unknown, could not be estimated, or was not applicable (because there are no coordinates).
georeferencedBy	A list (concatenated and separated) of names of people, groups, or organizations who determined the geo-reference for the location.
georeferenceProtocol	A description or reference for the methods used to determine the spatial footprint, coordinates, and uncertainties.
georeferenceSources	A list (concatenated and separated) of maps, gazetteers, or other resources used to geo-reference the location.
identifiedBy	A list (concatenated and separated) of names of people, groups, or organizations who assigned the taxon to the subject. If we did not know the name, we used the value “ND” *No determinado* (Not determined).
dateIdentified	The date on which the subject was identified as representing the taxon. Format yyyy-mm-dd. If we did not know the date, we used “9999”.
typeStatus	A list (concatenated and separated) of nomenclatural types applied to the subject. If the nomenclatural type did not apply, we used the value “NA” *No aplica* (Not applicable).
scientificName	The full scientific name of the lowest taxonomic rank determined.
originalNameUsage	The taxon name, as it originally appeared when first determined.
kingdom	The full scientific name of the kingdom in which the taxon is classified.
phylum	The full scientific name of the phylum in which the taxon is classified.
class	The full scientific name of the class in which the taxon is classified.
order	The full scientific name of the order in which the taxon is classified.
family	The full scientific name of the family in which the taxon is classified.
genus	The full scientific name of the genus in which the taxon is classified.
specificEpithet	The name of the species epithet of the scientificName.
infraspecificEpithet	The name of the lowest or terminal infraspecific epithet of the scientificName. If the infraspecific epithet did not apply, we used the value “NA” *No aplica* (Not applicable).
taxonRank	The taxonomic rank of the most specific name in the scientificName.
scientificNameAuthorship	The authorship information for the scientificName formatted according to the conventions.
taxonomicStatus	The status of the use of the scientificName as a label for a taxon.

## Dataset description

**Object name:** Darwin Core Archive CracidMex1: a comprehensive database of global occurrences of cracids (Aves, Galliformes) with distribution in Mexico

**Character encoding:** UTF-8

**Format and storage mode:** xlsx; ASCII csv, tab-delimited; decimal separator: ‘.’

**Distribution:** http://ipt.pensoft.net/ipt/resource.do?r=cracidmex1

**Publication date of data:** 2014-03-10

**Language:** Spanish.

**Metadata language:** English.

**Date of metadata creation:** 2014-01-08

**Hierarchy level:** Dataset
